# Application of Bayesian Additive Regression Trees for Estimating Daily Concentrations of PM_2.5_ Components

**DOI:** 10.3390/atmos11111233

**Published:** 2020-11-16

**Authors:** Tianyu Zhang, Guannan Geng, Yang Liu, Howard H. Chang

**Affiliations:** 1 Department of Biostatistics and Bioinformatics, Emory University, Atlanta, GA 30322, USA; 2 State Key Joint Laboratory of Environment Simulation and Pollution Control, School of Environment, Tsinghua University, Beijing 100084, China; 3 Gangarosa Department of Environmental Health, Emory University, Atlanta, GA 30322, USA

**Keywords:** regression trees, machine learning, Bayesian model, particulate matter, Community Multiscale Air Quality (CMAQ), aerosol optical depth

## Abstract

Bayesian additive regression tree (BART) is a recent statistical method that combines ensemble learning and nonparametric regression. BART is constructed under a probabilistic framework that also allows for model-based prediction uncertainty quantification. We evaluated the application of BART in predicting daily concentrations of four fine particulate matter (PM_2.5_) components (elemental carbon, organic carbon, nitrate, and sulfate) in California during the period 2005 to 2014. We demonstrate in this paper how BART can be tuned to optimize prediction performance and how to evaluate variable importance. Our BART models included, as predictors, a large suite of land-use variables, meteorological conditions, satellite-derived aerosol optical depth parameters, and simulations from a chemical transport model. In cross-validation experiments, BART demonstrated good out-of-sample prediction performance at monitoring locations (*R^2^* from 0.62 to 0.73). More importantly, prediction intervals associated with concentration estimates from BART showed good coverage probability at locations with and without monitoring data. In our case study, major PM_2.5_ components could be estimated with good accuracy, especially when collocated PM_2.5_ total mass observations were available. In conclusion, BART is an attractive approach for modeling ambient air pollution levels, especially for its ability to provide uncertainty in estimates that may be useful for subsequent health impact and health effect analyses.

## 1. Introduction

Ambient fine particulate matter pollution (PM_2.5_) is regulated worldwide because of its well-established associations with cardiorespiratory diseases and premature mortality [1]. PM_2.5_ pollution is a complex mixture of chemically and structurally diverse constituents, including elemental carbon (EC), organic carbon (OC), metals, and ions such as sulfate and nitrate. Monitoring networks for PM_2.5_ components are considerably sparser compared to other air pollutants, which contributes to the challenge of examining differential toxicity across PM_2.5_ components in population-based studies [2]. The ability to accurately estimate PM_2.5_ components at locations and at time points without monitoring data can help better support epidemiological studies analyses.

Various models have been developed to estimate concentrations of PM_2.5_ components using meteorological parameters, land-use variables, simulations from chemical transport models, and satellite-derived parameters. These include generalized additive models that allow for nonlinear associations [3], geostatistical models that incorporate spatial–temporal dependence [4], and machine learning algorithms such as random forest, neural networks, and ensemble modeling [5–8]. The main advantages of machine learning methods include the ability to handle large sets of highly correlated predictors, and the ability to construct complex predictive algorithms that are nonadditive and nonlinear.

However, one limitation of machine learning methods compared to model-based regression approaches is the lack of uncertainty quantification for individual predictions. To address this issue, in this paper, we examine the use of a recent statistical learning algorithm, Bayesian additive regression tree (BART) [9,10] for predicting PM_2.5_ components. BART employs a sum-of-trees approach, such that the prediction is based on contributions from many decision trees in a regression framework. More importantly, BART is a probabilistic model-based method that provides straightforward uncertainty quantification for predictions (e.g., via prediction standard error and prediction intervals), which are important for subsequent health impact and health effect analyses [11]. BART has been utilized in various prediction problems [12–14], but we are not aware of previous applications in modeling ambient air pollution.

We applied BART to four major PM_2.5_ components (EC, OC, nitrate, and sulfate) in California during the period 2005 to 2014 by reanalyzing data from a previous study that used random forest [8]. The main objective was to evaluate the prediction performance of BART and whether it can be tuned to achieve a balance between prediction accuracy and calibrated uncertainty intervals in cross-validation experiments. We also investigated the relative usefulness of numerical model simulations, satellite-derived aerosol optical depth (AOD), and total PM_2.5_ mass in predicting component concentrations. Understanding how different predictors contribute to prediction performance may help guide model development in other study settings.

## 2. Materials and Methods

### 2.1. Data Sources

The study area encompassed the state of California and an 80 km buffer from the state boundary. For the period 2005 to 2014, daily 24 h concentrations of total PM_2.5_ mass and its components OC, EC, sulfate, and nitrate from 55 monitors were obtained from the Chemical Speciation Network and the Interagency Monitoring of Protected Visual Environments (IMPROVE) Network. Data harmonization to account for different samplers and analytic methods between the two networks was described in Meng et al. [3]. Monitor locations are shown in Figure S1 (Supplementary Materials).

A spatial grid with the resolution of 1 km by 1 km was designed over the study area to spatially align predictors at different spatial resolutions. First, we obtained eight satellite-derived fractional AOD components (components 1, 2, 3, 6, 8, 14, 19, and 21) from the Multi-Angle Imaging Spectroradiometer (MISR) at 4.4 km spatial resolution. These fractional AOD components aim to reflect different particle shapes, scattering properties, and effective radius of the aerosol mixture. We also obtained (1) MISR fractional AOD based on aerosol particle properties (absorption, small, medium, large, and nonspherical AOD [15], (2) total AOD from MISR, and (3) total AOD from the Multi-Angle Implementation of Atmospheric Correction (MAIAC) algorithm at 1 km spatial resolution [16]. Each fractional AOD component aims to represent aerosols with different properties (e.g., particle diameter, shape, and refractive index), which are described in Table S1 (Supplementary Materials).

Additional predictors include (1) numerical model simulations for total PM_2.5_ mass, OC, EC, sulfate, and nitrate from the Community Multiscale Air Quality (CMAQ) model version 5.0.2 at a 12 km spatial resolution, (2) daily average temperature, precipitation, wind speed, solar radiation, potential evaporation, boundary layer height, and humidity from the North America Land Data Assimilation Systems Phase 2 at an approximately 13 km spatial resolution, and (3) several land-use variables (elevation, percent impervious surface, forest cover, shrub cover, and cultivated land), population density, and length of major roads, highways, and interstate freeways. Additional details on data sources and processing steps were given in Geng et al. [8] and are summarized in the Supplementary Materials.

### 2.2. Bayesian Additive Regression Trees

BART is a Bayesian regression approach that aims to model a response variable *y_i_* as a function of *P* predictors *x_i_* = (*x*_*i*1_, *x*_*i*2_, … , *x*_*i*p_) in a flexible manner that captures potential nonlinear relationships and complex interactions among predictors. This is accomplished by using a sum-of-trees framework. Specifically, a BART model with *K* trees is given by $y_{i}=\sum_{k=1}^{K}T_{k}\left(M_{k};x_{i}\right)+\varepsilon_{i},$, where *T_k_*(*M_k_*; *x_i_*) encodes a specific decision tree structure with a set of terminal nodes *M_k_* (also known as leaves) that are dependent on the predictor vector *x_i_*. The component *ε_i_* represents independent mean-zero normal error with variance *σ*^2^.

Each tree *T_k_*(*M_k_*; *x_i_*) contains a set of internal (nonterminal) nodes with binary splitting rules based on a single predictor in the form of *x_ij_* ≤ *c* or *x_ij_* > *c* for a threshold *c*. The decision process continues until a terminal node is reached and the observation is assigned the leaf value of this tree. The leaf parameter *M_k_* = (*μ*_1*k*_, *μ*_2*k*_, … , *μ_bk_*) gives the set of terminal values of the *k*-th tree with *b* terminal nodes. Hence, point predictions from BART can be interpreted as the sum of a specific terminal node from *K* trees.

### 2.3. Bayesian Inference

BART contains three stochastic components: the residual error *ε_i_* with variance *σ*^2^, the tree structures *T*_1_, … , *T_K_*, and the corresponding leaf node values *M*_1_, …, *M_K_*. One needs to construct probabilistic distributions that assign prior probability to all possible sum-of-trees. Independence is assumed a priori between trees, between leaf nodes conditioned on trees, and the residual variance as follows:

$$f\left(T_{1},\ldots ,T_{K},M_{1},\ldots ,M_{K},\sigma^{2}\right)=f\left(\sigma^{2}\right)\prod_{k=1}^{K}\prod_{l=1}^{b_{k}}f\left(\mu_{lk}|T_{k}\right)f\left(T_{k}\right).$$

The distribution of trees *f* (*T_k_*) is governed by its depth *d*, which has a prior distribution that is proportional to *α*(1 + *d*)^−*β*^ with *α* ∈ (0, 1) and *β* ∈ [0, ∞). Hence, larger values of *α* and *β* favor smaller trees, and these parameters can be tuned in cross-validation experiments. Terminal node values within a tree are assumed to follow a normal distribution with mean [*max*(*y*) – *min*(*y*)]/(2*K*) and variance $\sigma_{\mu }^{2}$. This is similar to the specification of a “ridge regression” for improving estimation accuracy.

Other probabilistic assumptions in the model include the following: at each internal node, each splitting predictor variable has equal prior probability of being chosen, i.e., with probability 1/*P*. Because of the sum-of-tree approach in BART, a large number of correlated variables may lead to model overfit and high collinearity. Recently, Linero [17] developed a method that allows for variable selection to impose sparsity, which we also considered in the analysis. Once the splitting variable is determined, the splitting value has equal probability from the set of unique values. Finally, the prior values for variance $\sigma_{\mu }^{2}$ and *σ*^2^ are assumed to follow noninformative inverse Gamma distributions.

BART can be fitted via Markov chain Monte Carlo (MCMC) algorithms that generate samples of all model parameters and predictions from their corresponding probability (posterior) distributions. Given *S* samples of each prediction, the point prediction is defined as the means of all posterior samples, and a 95% uncertainty/prediction interval is given by the 2.5th and the 97.5th quantiles of posterior samples. The MCMC algorithms can be efficiently carried using the package *BART* in the R statistical software [18]. Details for performing Bayesian inference for ensemble regression tress can be found elsewhere [19,20].

### 2.4 Application to California PM_2.5_ Component Modeling

We applied BART to predict daily concentrations of PM_2.5_, EC, OC, sulfate (SO_4_) and nitrate (NO_3_). Tuning parameters that control for the number of trees and the depth of the trees were adjusted to achieve the correct coverage probability for 95% posterior prediction intervals for in-sample data. All BART models were fitted with 8000 number of burn-in samples, and 2000 posterior samples were used for prediction.

We were interested in the importance of four types of predictors: total PM_2.5_ mass, CMAQ simulated PM_2.5_ components, fractional AOD components, and other AOD parameters (i.e., MAIAC/MISR total AOD and MISR aerosol particle properties). Models with the inclusion of different groups of the above four types of predictors were fitted to test their relative importance. The analysis of prediction performance with and without PM_2.5_ data is of particular interest because of the potential to leverage the larger PM_2.5_ monitoring network for estimating PM_2.5_ components. All BART models included longitude, latitude, land-use variables, and meteorology.

We considered three types of cross-validation (CV) experiments to assess out-of-sample prediction performance. In ordinary CV, 10% of data were randomly left out in each CV fold to evaluate prediction performance at locations with monitoring data. In spatial CV, 10% of monitors were randomly left out in each fold to evaluate performance at locations without monitoring data. Lastly, in spatial cluster CV, we first used k-means to group the monitors into 10 clusters on the basis of their longitude and latitude (Figure S2, Supplementary Materials); then, each cluster was left out in each fold to evaluate spatial prediction performance at regions without monitoring data and without nearby monitors. We used three evaluation criteria: the linear *R^2^* and the root-mean-square error (RMSE) between the left-out observations and predictions, and the empirical probability of the 95% prediction intervals capturing the left-out values. A model with well-calibrated uncertainty intervals will have an empirical probability close to 95%.

## 3. Results

Table 1 presents the out-of-sample prediction performance of a BART model including meteorology, land use, CMAQ simulations, and MISR fractional AOD with variable selection. We found that, in general, models without variable selection or with additional AOD did not improve performance (results in Table S2a–c, Supplementary Materials). The only exception was for OC in spatial cluster CV where adding other AOD parameters improved performance. For all PM_2.5_ components, prediction performance was poorer for spatial prediction compared to prediction at locations with monitoring data. In spatial CV, *R^2^* was highest for SO_4_ and lowest for EC, which can be explained by the higher and lower spatial heterogeneity associated with these two pollutants. PM_2.5_ total mass is an important variable for predicting PM_2.5_ components. Particularly, we saw the largest improvement in prediction associated with OC. When PM_2.5_ was included as a predictor, CV *R^2^* was highest for SO_4_ and OC, and lowest for EC. This is likely due to SO_4_ and OC being the two major constituents of PM_2.5_ by mass in the study region.

From Table 1, the 95% prediction interval coverage also showed excellent performance in ordinary CV and continued to achieve close to 95% coverage for spatial predictions. RMSE and R^2^ when using default BART settings are given in Table S3 (Supplementary Materials). We found that, when using the default settings of prior distributions and 200 trees, the models showed evidence of overfitting as the 95% prediction intervals had lower coverage probability than desired (78% to 92%). This under-coverage was likely due to an underestimation of the true residual variability in the model. However, when we reduced the number of trees and decreased the depth of trees, we could achieve a better 95% coverage probability, usually sacrificing little *R^2^* (0.02 to 0.05). In some cases, *R^2^* improved further with tuning, especially in predicting at locations without monitors (e.g., spatial CV for NO_3_ where *R^2^* improved from 0.52 to 0.59).

Table 2 gives posterior estimates of two key BART parameters: $\sigma_{\mu }^{2}$ (variance of terminal nodes) and *σ*^2^ (residual variance). First, we noted that the residual error *σ*^2^ decreased considerably when PM_2.5_ total mass was included as a predictor. This indicates that the BART ensemble trees were able to better explain variations in PM_2.5_ species, which may explain the better prediction performance observed in Table 1. Second, $\sigma_{\mu }^{2}$ reflected differences in variability in PM_2.5_ component concentrations and followed the same order of observed standard deviation for the four components: 3.28 for OC, 2.59 for NO_3_, 1.08 for SO_4_, and 0.74 for EC.

**Figure 1.**
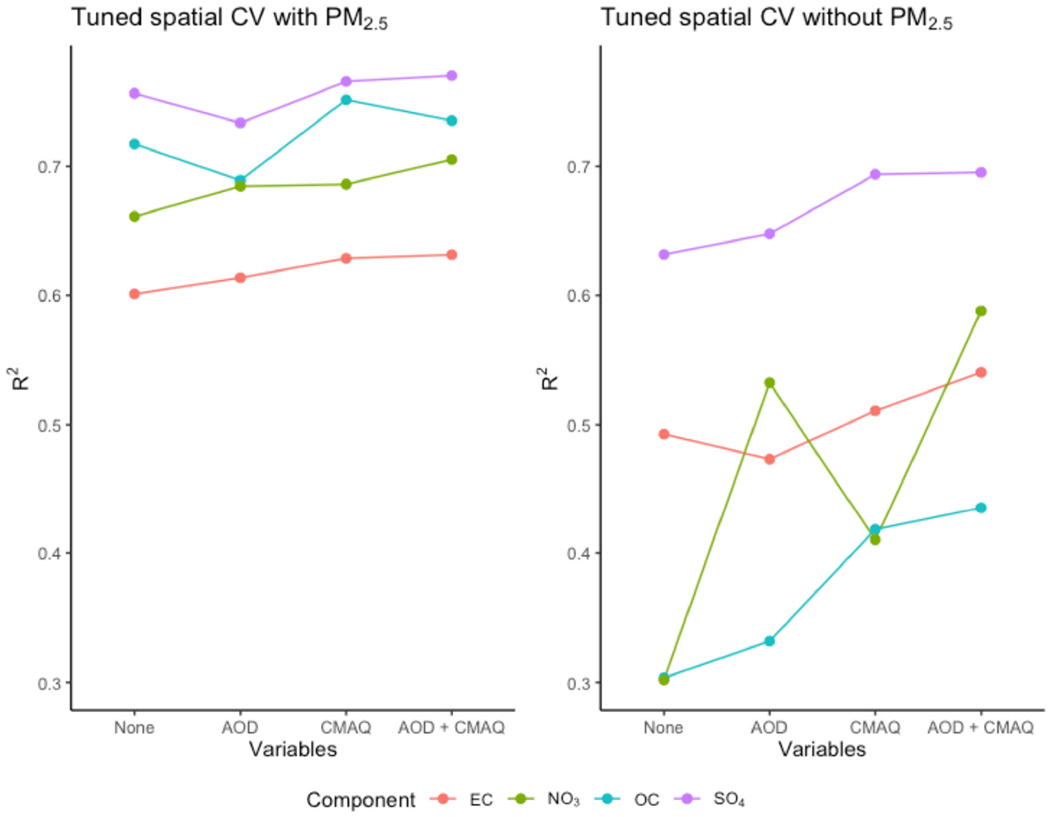
*R*^2^ of spatial cross-validation (CV) results for predicting PM_2.5_ components elemental carbon (EC), organic carbon (OC), sulfate (SO_4_), and nitrate (NO_3_), comparing the inclusion of only Multi-Angle Imaging Spectroradiometer (MISR) fraction AOD, only CMAQ simulations, or both AOD and CMAQ. All models contain meteorology and land-use variables.

Figure 1 demonstrates the usefulness of including AOD parameters or CMAQ simulations in the set of predictors. In spatial CV, including AOD parameters or CMAQ simulations could improve *R^2^*, especially when PM_2.5_ was not included as a predictor. However, including PM_2.5_ as a predictor resulted in greater *R^2^* improvement compared to including AOD and/or CMAQ. AOD parameters were most useful for predicting NO_3_ when PM_2.5_ was not included as a predictor. Prediction performance for SO_4_ and EC depended less on the inclusion of PM_2.5_, AOD, and CMAQ compared to other pollutants. Similar observations were found for RMSE, other CV experiments, and BART fitted with default settings. We tuned BART to have the desired 95% interval coverage. The resulting spatial CV predictions all had coverage above 90% regardless of the set of predictors used.

**Figure 2.**
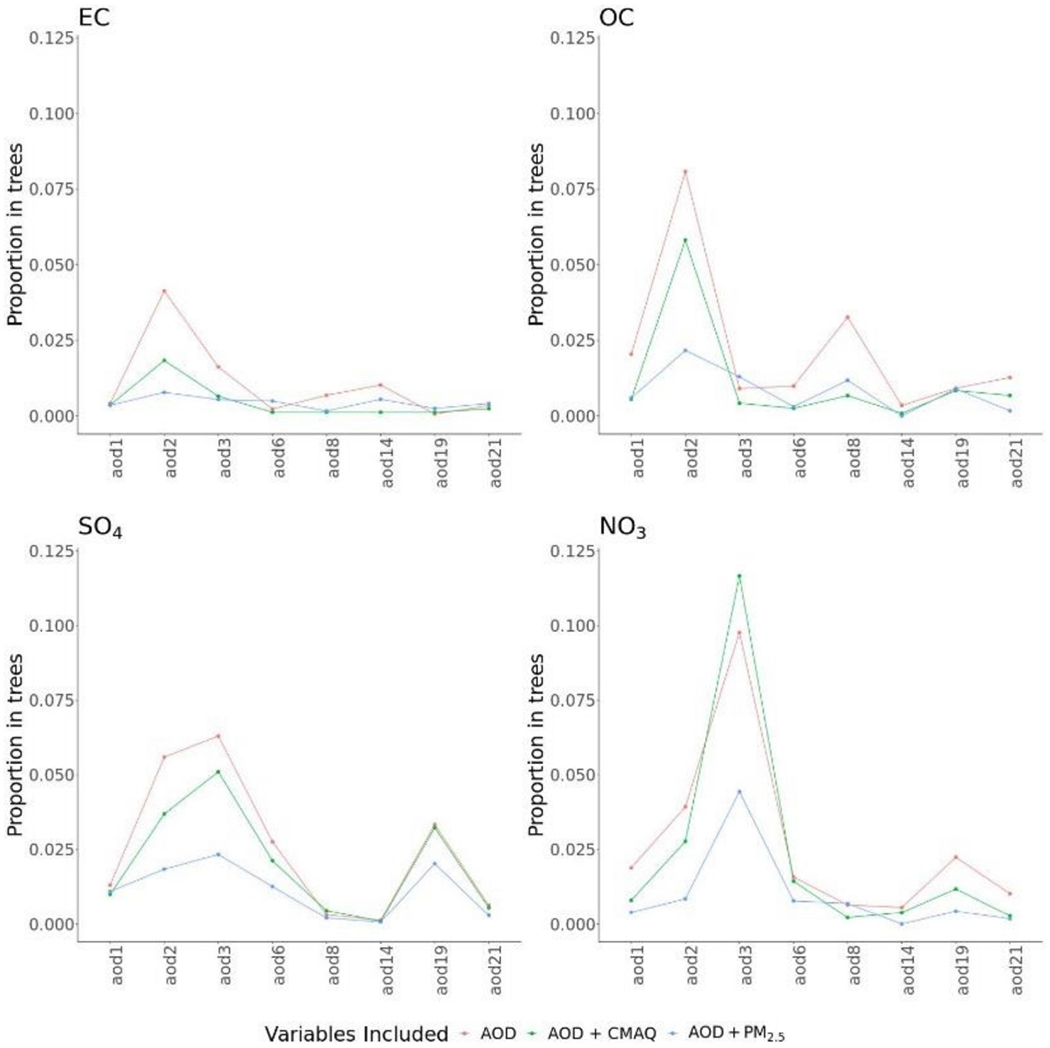
BART variable importance (proportion in trees) of individual AOD fractional components for predicting PM_2.5_ components elemental carbon (EC), organic carbon (OC), sulfate (SO_4_), and nitrate (NO_3_), under different predictor sets (with MISR fractional AOD, with AOD and CMAQ simulations, or with AOD and PM_2.5_ total mass). All models include meteorology and land-use predictors.

Figure 2 describes the importance for AOD parameters in different BART models with and without the presence of other predictors. Here, variable importance was measured by calculating the number of times a variable was used for splitting nodes across MCMC iterations. The pattern of variable importance was robust in models with only AOD, with AOD and CMAQ, or with AOD and PM_2.5_. However, the relative importance of different AOD parameters varied across PM_2.5_ components. For predicting NO_3_, AOD3 was highly important, and, for OC, AOD2 was the most important, followed by AOD8. For SO_4_, many AOD parameters showed moderate importance.

The importance for CMAQ simulations in different BART models with and without the presence of other predictors is given in Figure S3 (Supplementary Materials). Similar to AOD, the pattern of variable importance for CMAQ was robust across models. All PM_2.5_ components depended on CMAQ heavily, specifically on the corresponding pollutant (i.e., the CMAQ simulation for EC had the highest importance for predicting EC concentration). Generally, including PM_2.5_ reduced the importance of CMAQ simulations.

Variance importance measures of all predictors are given in Figure S4 (Supplementary Materials). For both EC and OC, the percentage impervious surface was the most important predictor as it is a proxy of urbanicity. Other important meteorological and geospatial predictors (ranked among the top five) included solar radiation for EC, OC, and sulfate, boundary layer height for EC and nitrate, population density for EC, and percentage forest cover for OC.

One advantage of the regression-based framework of BART is its ability to estimate the marginal effect (also known as partial dependence function) of a predictor of interest while accounting for all other predictors in the model. For example, the marginal effect of an AOD parameter at a specific value is defined as the average predicted PM_2.5_ component concentrations of all observations with that specific AOD value. For each PM_2.5_ component, the marginal effects of the most important fractional AOD parameter and the corresponding CMAQ simulations are given in Figure S5 (Supplementary Materials). We observed clear positive marginal associations between these predictors and PM_2.5_ component concentration with some evidence of nonlinearity at the low and high values of AOD.

**Figure 3.**
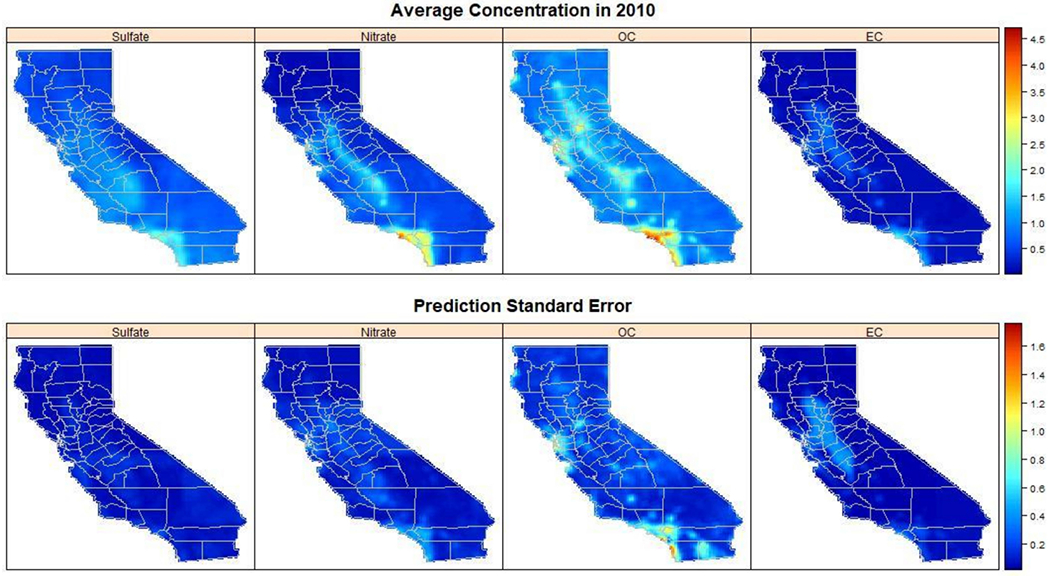
Estimated 2010 annual average of elemental carbon (EC), organic carbon (OC), sulfate, and nitrate in California. The prediction standard errors are for the annual averages. Concentration is given in μg/m^3^.

Figure 3 shows the estimated annual pollution concentrations over California for the year 2010, as well as the corresponding uncertainty measured as the average prediction standard error. Higher pollution concentrations were estimated in the central valley and southern California, particularly in the Los Angeles metropolitan area. Prediction standard errors also showed spatial variation and were largest for OC and EC, especially at regions with estimated high concentrations. Geng et al. [8] found similar spatial patterns in the four PM_2.5_ components during the study period 2005 to 2014

## 4. Discussion

Our study showcased the application of BART in modeling ambient air pollution. BART has received increasing attention in machine learning and statistical research because it borrows strengths from both modeling paradigms. Our analysis was motivated by BART’s ability to handle a large number of predictors via variable selection, complex interactions via the additive tree structure, and model-based uncertainty quantification via a Bayesian estimation procedure. The recent R package for fitting BART will further encourage its use in wider applications.

The BART methodology shares some similarities with two commonly used ensemble machine learning approaches that have been applied in estimating ambient air pollution, namely, random forest [21] and gradient boosting [22]. Random forest is an ensemble approach that averages predictions from many classification trees obtained by bootstrapping the original dataset and subsampling the set of predictors. Gradient tree boosting is a sequential procedure that repeatedly fits regression trees or other weak learners on the residuals from the previous step such that the resulting overall predictions can be interpreted as a weighted sum of predictions from multiple trees. Current implementations of gradient boosting further incorporate resampling of observations [23]. Both random forest and gradient tree boosting are frequently used in predicting ambient air pollution including fine and coarse particulate matter, ozone, and NO_2_ across different regions of the world [24,25].

While both random forest and gradient boosting utilize the concepts of combining predictions from many trees similar to BART, predictions from these two methods are not based on a regression model with probabilistic components. Specifically, the lack of a residual variance *σ*^2^ and the use of various tuning parameters to favor smaller trees and prevent overfitting make uncertainty quantification in the resulting predictions challenging [26,27]. The main advantage of BART is that it is constructed such that key components, i.e., tree structure *T_k_* and leaf nodes *M_k_*, are treated as unknown parameters. These parameters are estimated jointly in a single Bayesian hierarchical model such that prediction uncertainties can be easily calculated. The ability to modify distributional assumptions on various model parameters provides an opportunity to consider various model extensions, e.g., for high-dimensional predictors [28] and for random effect models [29].

We reanalyzed data from a recent study that utilized random forest [8]. We obtained similar prediction performance for all pollutants at locations with monitoring data. For spatial cluster prediction, our best models performed similarly for EC (*R*^2^ of 0.51 versus 0.53) and nitrate (*R*^2^ of 0.50 versus 0.48), poorer for OC (*R*^2^ of 0.39 versus 0.48), and better for sulfate (*R*^2^ of 0.65 versus 0.52). Some of the difference could be attributed to reductions in accuracy to ensure that the prediction intervals had the desired probabilistic property. The variable importance measure from BART is defined differently than for random forest, but we identified similar important fractional AOD parameters to Geng et al. [8]. Specifically, AOD2 and AOD3 were the most significant AOD components, possibly due to their similar sizes to those of PM_2.5_ species. Light-absorbing components, AOD8 and AOD14, were also important predictors in EC and OC models; however, other nonabsorbing components also contributed to the model, probably because AOD8 and AOD14 were not sensitive enough to provide the spatial variability. The significance of AOD19 in the sulfate and nitrate models could be caused by particles with the dust component size distributions and shapes in California.

The main advantage of BART is its ability to provide uncertainty measures that can vary across space, time, and predictor values. These uncertainties can be used in health impact assessments using Monte Carlo methods [30–33], as well as in subsequent health effect analyses [33–35]. Previous studies found that incorporating uncertainties in exposures usually led to larger intervals for health effect estimates.

One limitation of our BART model is that it did not explicitly account for spatial autocorrelation in ambient air pollution concentration. Although geographic information such as latitude and longitude can be used as predictors, BART may not capture small-scale spatial dependence in the outcome. Previous analyses using machine learning methods considered introducing spatial dependence via spatially smoothed pollutant concentration as a predictor [5,36]. We were not able to perform a detailed examination for BART due to the sparse monitoring network for PM_2.5_ components. However, this approach may not be optimal for spatial interpolation [37] and makes assessing prediction uncertainty more challenging because observed concentrations are also used as predictors.

Another limitation is that our training and prediction datasets were restricted to locations and days when both MISR AOD retrievals and PM_2.5_ component measurements were available. The average number of MISR retrievals is around 30 per year across grid cells, and regulatory monitors typically provide measurements every 6 days. To gap-fill predictions, recent studies for total PM_2.5_ mass considered imputing total AOD that was informatively missing [38,39] and ensemble modeling that included members without AOD as a predictor [40]. Both approaches warrant further investigation for predicting PM_2.5_ components and for BART.

## 5. Conclusions

BART is an attractive approach for developing flexible prediction models for ambient air pollution concentrations. In particular, BART offers the ability to provide uncertainty in estimates that may be useful for subsequent health impact and health effect analyses. In our California case study, major PM_2.5_ components could be estimated with good accuracy, especially when collocated PM_2.5_ total mass observations were available.

## Supplementary Material

Supplementary materials

## Figures and Tables

**Table 1.** Tenfold ordinary, spatial, and spatial cluster cross-validation (CV) results using Bayesian additive regression trees (BARTs) for predicting fine particulate matter (PM_2.5_) components elemental carbon (EC), organic carbon (OC), sulfate (SO_4_), and nitrate (NO_3_), with and without using PM_2.5_ total mass as a predictor. All models include meteorology, land-use variables, Community Multiscale Air Quality (CMAQ) simulations, and fractional aerosol optical depth (AOD) with variable selection implemented.

		Without PM_2.5_			With PM_2.5_		

		*R*^2^	RMSE	Cvg_95_	*R*^2^	RMSE	Cvg_95_
**Ordinary CV**	EC	0.67	0.42	0.95	0.78	0.35	0.95
	OC	0.62	1.84	0.96	0.84	1.18	0.95
	SO_4_	0.73	0.56	0.95	0.80	0.49	0.96
	NO_3_	0.65	1.53	0.95	0.80	1.17	0.95

**Spatial CV**	EC	0.54	0.50	0.93	0.63	0.45	0.93
	OC	0.44	2.26	0.93	0.74	1.54	0.92
	SO_4_	0.70	0.59	0.95	0.77	0.52	0.95
	NO_3_	0.59	1.66	0.95	0.71	1.40	0.93

**Spatial Cluster CV**	EC	0.51	0.52	0.94	0.64	0.44	0.93
	OC	0.27	2.66	0.91	0.69	1.66	0.91
	SO_4_	0.61	0.67	0.93	0.70	0.58	0.93
	NO_3_	0.50	1.83	0.93	0.72	1.38	0.93

RMSE: root-mean-square error; Cvg_95_: empirical coverage probability of the 95% prediction intervals.

**Table 2.** Posterior mean and 95% posterior interval of BART variance parameters from models with and without PM_2.5_ as a predictor. Parameter $\sigma_{\mu }^{2}$ describes the variability in terminal nodes across trees and *σ*^2^ describes the residual variability not explained by the ensemble trees.

	Without PM_2.5_		With PM_2.5_	

	$\sigma_{\mu }^{2}$	*σ*^2^	$\sigma_{\mu }^{2}$	*σ*^2^
EC	0.19 (0.15, 0.23)	0.14 (0.13, 0.14)	0.19 (0.17, 0.23)	0.09 (0.09, 0.09)
OC	8.88 (8.07, 10.5)	2.21 (2.17, 2.25)	5.01 (4.50, 5.54)	0.91 (0.90, 0.93)
SO_4_	0.51 (0.43, 0.62)	0.23 (0.22, 0.24)	0.35 (0.39, 0.46)	0.17 (0.16, 0.17)
NO_3_	7.07 (6.26, 8.21)	1.08 (1.06, 1.11)	7.32 (6.64, 8.75)	0.71 (0.70, 0.72)
