# Supplementary material for: Application of Bayesian Additive Regression Trees for Estimating Daily Concentrations of PM2.5 Components
Source: Atmosphere (Basel). Author manuscript; Available in PMC 2021 Jul 27. (PMC8315111; doi:10.3390/atmos11111233)
Supplement: Supplementary materials [file NIHMS1654077-supplement-Supplementary_materials.docx]

**Online Supplementary Materials for**

**Application of Bayesian Additive Regression Trees for Estimating Daily Concentrations of PM_2.5_ Components**

Tianyu Zhang,^1^ Guannan Geng,^2,3^ Yang Liu,^2^ Howard H. Chang^1,2^*

Department of Biostatistics and Bioinformatics, Emory University, Atlanta GA 30322

Department of Environmental Health, Emory University, Atlanta GA 30322

State Key Joint Laboratory of Environment Simulation and Pollution Control, School of Environment, Tsinghua University, Beijing 100084, People’s Republic of China

Email: [howard.chang@emory.edu](mailto:howard.chang@emory.edu)

**Table S1.** The eight MISR fractional AOD components (AOD1, AOD 2, AOD 3, AOD 6, AOD 8, AOD 14, AOD 19 and AOD 21 used in our analysis represent different particle characteristics of size, shape and complex refractive index). The eight fractional AOD components are named starting with particle shape, followed by a qualitative scattering property designation and ending with the effective radius for a number-weighted log-normal distribution. Single scattering albedo at 558 nm wavelength is added when necessary to distinguish components.

| Fractional AOD | Aerosol components | Name | d_min_^1^ | d_max_^1^ | d_c_^1^ | Single scattering albedo (558nm) |
| --- | --- | --- | --- | --- | --- | --- |
| AOD1 | Particle1 | Spherical_nonabsorbing_0.06 | 0.002 | 0.8 | 0.06 | 1 |
| AOD2 | Particle 2 | Spherical_nonabsorbing_0.12 | 0.002 | 1.5 | 0.12 | 1 |
| AOD3 | Particle 3 | Spherical_nonabsorbing_0.26 | 0.02 | 3.0 | 0.24 | 1 |
| AOD6 | Particle 6 | Spherical_nonabsorbing_2.8 | 0.2 | 100 | 1.00 | 1 |
| AOD8 | Particle 8 | Spherical_absorbing_0.12_ssa_green_0.9 | 0.002 | 1.5 | 0.12 | 0.9 |
| AOD14 | Particle 14 | Spherical_absorbing_0.12_ssa_green_0.8 | 0.002 | 1.5 | 0.12 | 0.8 |
| AOD19 | Particle 19 | Grains_mode1_h1 (dust) | 0.2 | 2.0 | 1.00 | 0.98 |
| AOD21 | Particle 21 | spheroidal_mode2_h1 (dust) | 0.2 | 12.0 | 2.00 | 0.90 |

^1^d_min_: the minimum particle diameters for a given aerosol component;

^2^d_max_: the maxmum particle diameters for a given aerosol component;

^3^d_c_: the characteristic diameter of the lognormal size distribution.

**Table S2. Comparison with alternative BART models.** 10-fold ordinary, spatial, and spatial-cluster cross-validation (CV) for predicting PM_2.5_ component elemental carbon (EC), organic carbon (OC), sulfate (SO_4_) and nitrate (NO_3_). All models include meteorology, land use, and CMAQ simulations. RMSE: root mean square error; Cvg_95_: empirical coverage probability of the 95% prediction intervals. Cases when alternatives model perform better than results in the primary model (Table 1) are highlighted

**S2-(a) Fractional AOD only without variable selection**

**Without PM_2.5_ With PM_2.5_**

**R^2^ RMSE Cvg_95_ R^2^ RMSE Cvg_95_**

**Ordinary CV** EC 0.68 0.42 0.95 0.77 0.35 0.95

OC 0.61 1.87 0.96 0.83 1.24 0.95

SO_4_ 0.73 0.56 0.95 0.79 0.49 0.96

NO_3_ 0.68 1.46 0.96 0.71 1.38 0.92

**Spatial CV**  EC 0.51 0.52 0.93 0.60 0.47 0.91

OC 0.45 2.26 0.93 0.71 1.61 0.89

SO_4_ 0.70 0.59 0.95 0.75 0.53 0.95

NO3 0.53 1.78 0.95 0.78 1.22 0.95

**Spatial Cluster CV** EC 0.49 0.53 0.93 0.64 0.44 0.93

OC 0.25 2.71 0.91 0.65 1.78 0.92

SO_4_ 0.65 0.64 0.94 0.73 0.56 0.94

NO_3_ 0.49 1.86 0.93 0.71 1.38 0.93

**S2-(b) Fractional AOD, MISR and MAIAC total AOD and MISR aerosol properties without variable selection**

**Without PM_2.5_ With PM_2.5_**

**R^2^ RMSE Cvg_95_ R^2^ RMSE Cvg_95_**

**Ordinary CV** EC 0.64 0.44 0.95 0.76 0.36 0.95

OC 0.54 1.98 0.96 0.81 1.28 0.95

SO_4_ 0.73 0.58 0.95 0.79 0.51 0.96

NO_3_ 0.69 1.48 0.94 0.74 1.37 0.95

**Spatial CV**  EC 0.51 0.52 0.93 0.63 0.45 0.93

OC 0.32 2.48 0.93 0.71 1.59 0.91

SO_4_ 0.66 0.65 0.94 0.76 0.54 0.95

NO_3_ 0.52 1.84 0.95 0.62 1.66 0.96

**Spatial Cluster CV** EC 0.48 0.54 0.93 0.58 0.49 0.93

OC 0.39 2.28 0.93 0.69 1.63 0.92

SO_4_ 0.61 0.71 0.92 0.70 0.61 0.93

NO_3_ 0.45 1.99 0.92 0.66 1.55 0.92

**S2-(c) Fractional AOD, MISR and MAIAC total AOD and MISR aerosol properties with variable selection implemented**

**Without PM_2.5_ With PM_2.5_**

**R^2^ RMSE Cvg_95_ R^2^ RMSE Cvg_95_**

**Ordinary CV** EC 0.66 0.43 0.95 0.74 0.38 0.95

OC 0.61 1.81 0.96 0.79 1.35 0.94

SO_4_ 0.74 0.57 0.96 0.78 0.52 0.95

NO_3_ 0.59 1.71 0.95 0.81 1.17 0.95

**Spatial CV**  EC 0.45 0.56 0.92 0.58 0.49 0.93

OC 0.44 2.18 0.94 0.71 1.56 0.92

SO_4_ 0.68 0.63 0.94 0.76 0.54 0.94

NO_3_ 0.55 1.80 0.95 0.69 1.49 0.93

**Spatial Cluster CV** EC 0.47 0.54 0.93 0.59 0.48 0.93

OC 0.36 2.33 0.93 0.73 1.51 0.93

SO_4_ 0.59 0.72 0.92 0.72 0.58 0.94

NO_3_ 0.47 1.94 0.92 0.67 1.54 0.92

**Table S3. Comparison with the primary BART model without tuning.** 10-fold ordinary, spatial, and spatial-cluster cross-validation (CV) results using Bayesian Additive Regression trees (BART) for predicting PM_2.5_ component elemental carbon (EC), organic carbon (OC), sulfate (SO_4_) and nitrate (NO_3_), with and without using PM_2.5_ total mass as a predictor. All models include meteorology, land use variables, CMAQ simulations, and fractional AOD with variable selection implemented. RMSE: root mean square error; Cvg_95_: empirical coverage probability of the 95% prediction intervals. Cases when alternatives model perform better than results in the primary model (Table 1) are highlighted

**Without PM_2.5_ With PM_2.5_**

**R^2^ RMSE Cvg_95_ R^2^ RMSE Cvg_95_**

**Ordinary CV** EC 0.72 0.39 0.88 0.81 0.33 0.87

OC 0.67 1.72 0.91 0.85 1.15 0.89

SO4 0.77 0.51 0.92 0.83 0.44 0.92

NO3 0.66 1.51 0.89 0.79 1.20 0.88

**Spatial CV**  EC 0.53 0.52 0.87 0.58 0.51 0.78

OC 0.45 2.34 0.85 0.71 1.62 0.82

SO4 0.73 0.56 0.90 0.78 0.51 0.89

NO3 0.52 1.80 0.84 0.70 1.42 0.86

**Spatial Cluster CV** EC 0.54 0.51 0.85 0.65 0.45 0.78

OC 0.34 2.61 0.87 0.74 1.54 0.86

SO4 0.72 0.57 0.90 0.80 0.48 0.91

NO3 0.59 1.66 0.87 0.70 1.44 0.86

**Figure S1.** Locations of 55 PM_2.5_ component monitors.

**
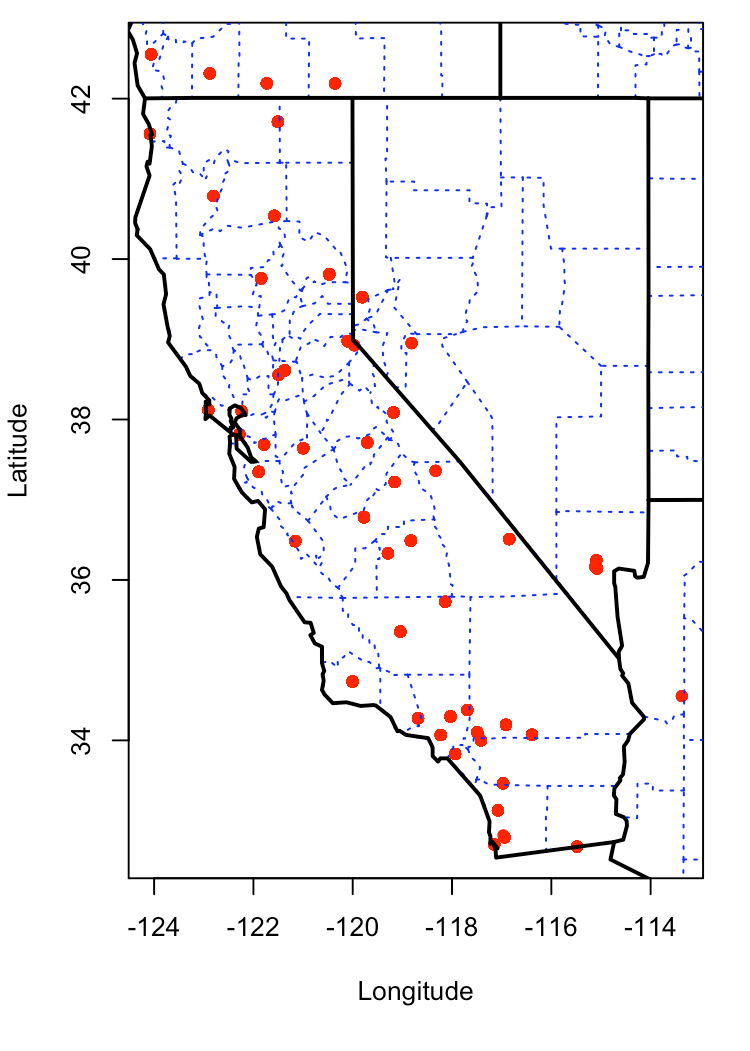
**

**Figure S2.** Locations of monitoring data and test-group for the 10-fold spatial-cluster cross-validation experiment.


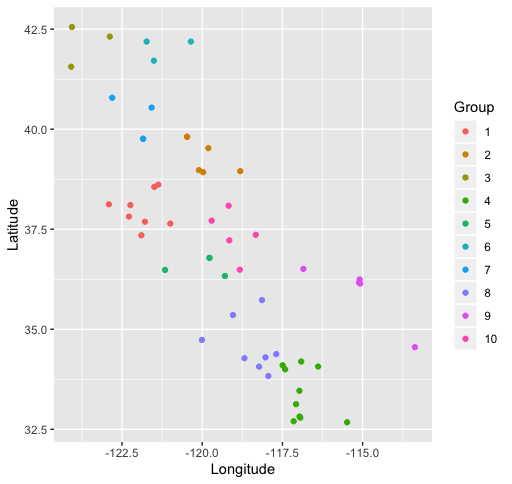


**Figure S3.** BART Variable importance (proportion in trees) of individual CMAQ simulations for predicting PM_2.5_ component elemental carbon (EC), organic carbon (OC), sulfate (SO_4_) and nitrate (NO_3_), under different predictor sets (with MISR fractional AOD, with AOD and CMAQ simulations, with AOD and PM_2.5_ total mass). All models include meteorology and land use predictors.


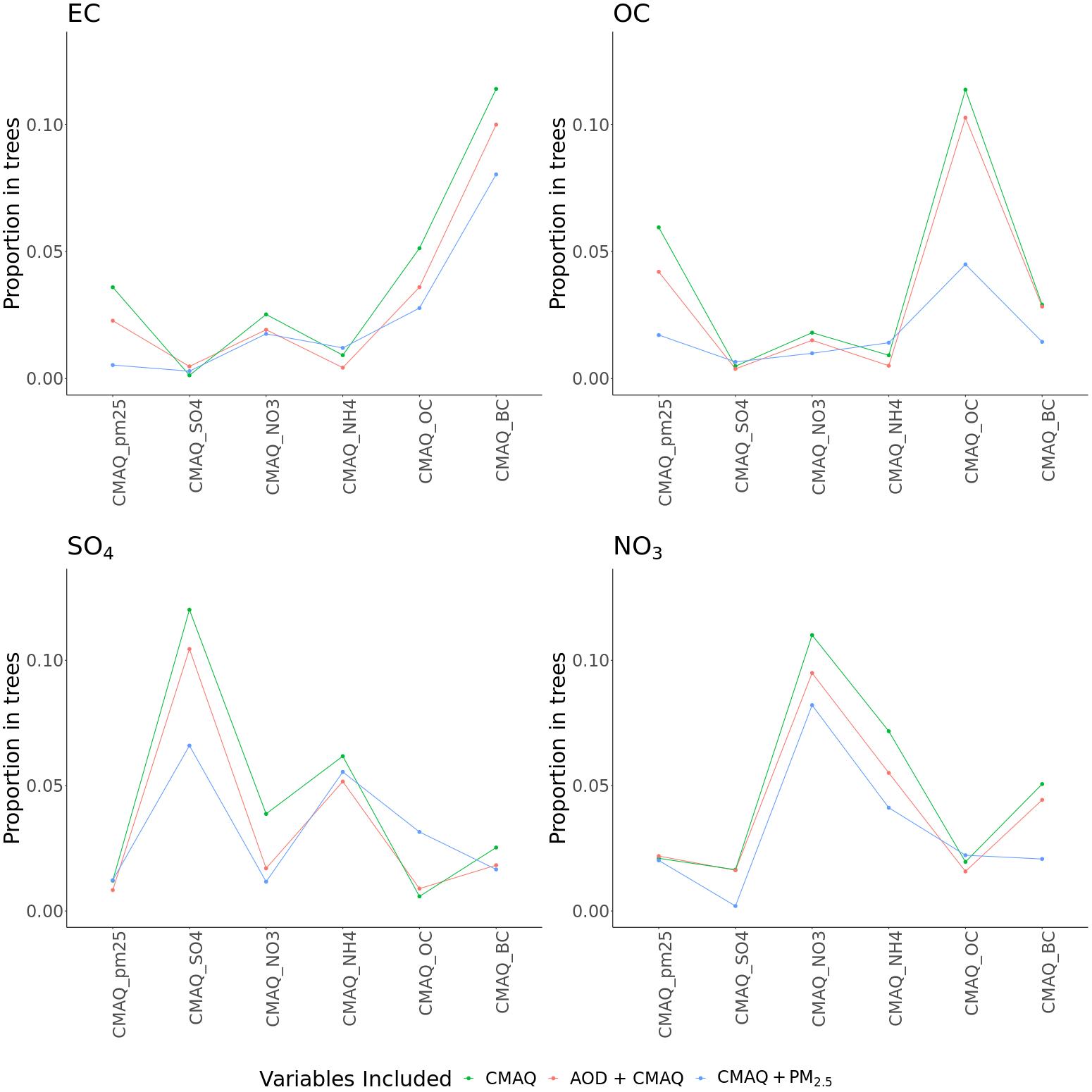


**Figure S4.** BART variable importance (proportion in trees) for all variables, including fraction AOD parameters, CMAQ model simulation, land use variables and meteorology.


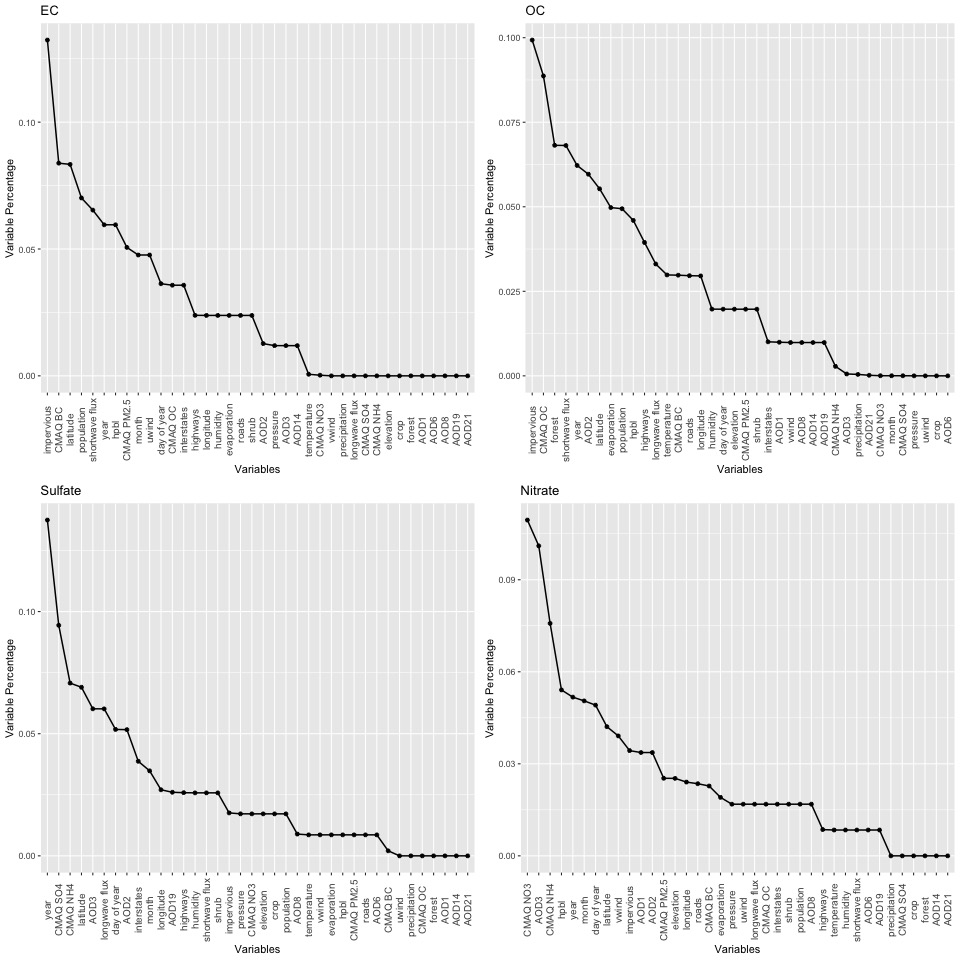


**Figure S5.** Marginal effects of the most important fractional AOD parameter and CMAQ simulation for each PM_2.5_ component. Marginal effects are evaluated over a range defined as the 5^th^ and 95^th^ percentiles of observed predictor values.


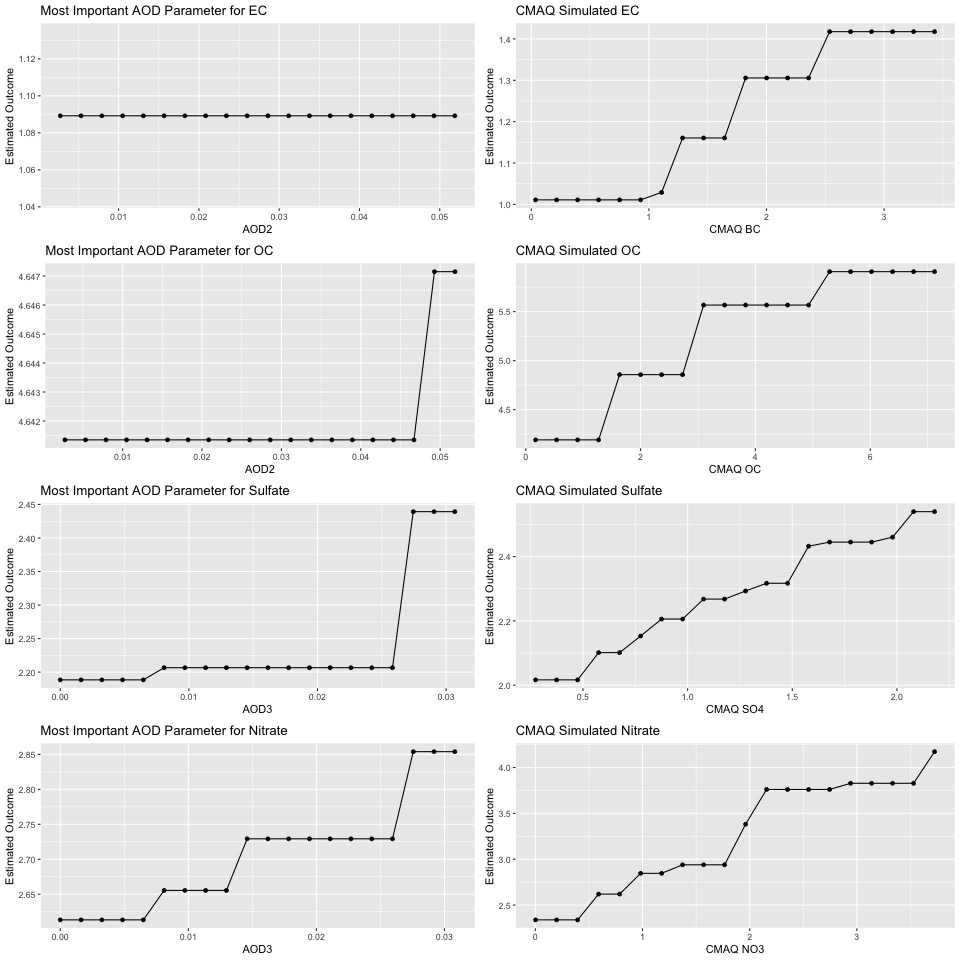


**Section A: Data Source and Processing for Predictors**

We obtained satellite-derived AOD from Multi-angle Imaging SpectroRadiometer (MISR). MISR simultaneously retrieves data from nine different angles, which provides data to distinguish the aerosol particles. We downloaded Aerosol Data V23 level 2 for the years 2005-2014 from the NASA Earthdata portal (https://search.earthdata.nasa.gov/) which contains 74 different aerosol components. In addition, eight tractional AOD components (i.e., component 1, 2, 3, 6, 8, 14, 19 and 21) were developed to represent the different particle shapes, scattering properties and effective radius for a log-normal distribution. We used the following equation to convert any MISR aerosol observation to the fractional AOD components:

$$\text{AOD }\text{i}\text{ = }\frac{\sum_{\text{j}\text{=1}}^{\text{74}} \text{α}\text{AOD}_{\text{mixture j}}\text{×}\text{Fraction}_{\text{component }\text{i}\text{ in mixture }\text{j}}}{\text{Number of successful mixtures}}$$

where $\text{AOD}_{\text{mixture j}}$ is the AOD mixture *j*; $\text{Fraction}_{\text{component }\text{i}\text{ in mixture }\text{j}}$ is the contribution of component *i* to the AOD for mixture *j*; if mixture *j* is retrieved successfully, then *α*=1, otherwise *α*=0. We also considered different sums of the 8 AOD components for absorbing, non-absorbing, spherical and non-spherical particles.

The numerical model simulations used in this study were based on the Community Multiscale Air Quality (CMAQ) model version 5.02 which used meteorological conditions from the Meteorological Research and Forecast (WRF) model version v3.4. Details of the model configuration for WRF and CMAQ can be found in (Zhang et al. 2019). The National Land Cover Database (NLCD) was used as the input to the WRF model, and the Meteorology-Chemistry Interface Processor version 4.1.3 was used to generate the input to the atmospheric parameters in CMAQ model. The chemical boundary conditions for the CMAQ model were derived from the annual-specific simulation of the global GEOS-Chem model. Anthropogenic emissions inputs were based on data from 2005, 2008 and 2011 National Emissions Inventories.

Daily temperature, wind speed and humidity data for the spatial resolution of approximately 13 km were obtained from the North America Land Data Assimilation Systems phase 2 (NLDAS-2, http://ldas.gsfc.nasa.gov/nldas/). Meteorological data were all averaged between 9:00 am to 12:00 pm and interpolated to the 1km grid cells by inverse-distance weighting.

Elevation data were based on the Advanced Spaceborne Thermal Emission and Reflection Radiometer (ASTER) Global Digital Elevation Map (GDEM) (<https://asterweb.jpl.nasa.gov/gdem.asp>) version 2.

Data on road networks were obtained from ESRI StreetMap USA (Environmental Systems Research Institute, Inc., Redlands, CA). Impervious surface, forest cover, shrub cover and cultivated land cover information at 30 m spatial resolution were taken from NLCD (https://www.mrlc.gov) for the year 2006 and 2011. Population data at 1 km spatial resolution were extracted from the LandScan Global Population Database (<https://landscan.ornl.gov/>). Elevation, impervious surface, forest cover, shrub cover and cultivated land cover were averaged while population and road lengths were summed within each 1-km grid cell.

Zhang, Yuqiang, Kristen M. Foley, Donna B. Schwede, Jesse O. Bash, Joseph P. Pinto, and Robin L. Dennis. 2019. “A Measurement-Model Fusion Approach for Improved Wet Deposition Maps and Trends.” Journal of Geophysical Research: Atmospheres 124 (7): 4237–51. https://doi.org/10.1029/2018JD029051.
